# Defect closure after duodenal endoscopic submucosal dissection for a tumor of the minor papilla in a patient with pancreas divisum: reopenable-clip over-the-line method

**DOI:** 10.1055/a-2625-3764

**Published:** 2025-07-10

**Authors:** Tatsuma Nomura, Makoto Kobayashi, Takanobu Mitani, Yuto Ikadai, Hiroaki Kumazawa, Yoshiaki Isono, Katsumi Mukai

**Affiliations:** 1 36951 Department of Gastroenterology, Suzuka General Hospital, Suzuka, Japan; 2 36951 Department of Endoscopy Center, Suzuka General Hospital, Suzuka, Japan; 3 37036 Department of Gastroenterology, Yokkaichi Municipal Hospital, Yokkaichi, Japan

Closure of large duodenal endoscopic submucosal dissection (ESD) defects that include the entire minor papilla are extremely difficult because the duodenal muscle layer on the pancreatic side is relatively hard and difficult to maneuver 1 . We overcame these difficulties by devising a method called the reopenable-clip over-the-line method (ROLM), which facilitates the closure of large defects even in narrow lumens 2 3 . Herein, we present a case of defect closure after duodenal ESD for a minor papillary tumor with pancreatic divisum using the ROLM.

The patient in this case had early duodenal cancer ( Fig. 1 , Video 1 ). The lesion was approximately 40 mm in size and covered the entire minor papilla. Magnetic resonance cholangiopancreatography revealed partial pancreas divisum; therefore, it was considered important to avoid obstructing the flow of pancreatic juice from the accessory pancreatic duct. En bloc resection of the tumor was initially performed using ESD. In the expectation of bleeding due to the large size of the tumor, dual-channel rapid hemostasis was performed using a gas-free immersion system, and the procedure was performed with saline immersion 4 . Following resection, the defect measured approximately 50 mm, with the opening of the accessory pancreatic duct located at the defect edge. A duodenoscope was used to place the pancreatic duct stent in the duodenum. Finally, defect closure was performed using the ROLM, ensuring that the pancreatic duct stent was not embedded in the ulcer floor by securing the closure with clips. Using a 4-mm tapered hood, the pancreatic duct stent was positioned on the side of the duodenal lumen, without embedding it into the ulcer floor, and the defect edge on the opposite side was grasped with the clip 5 . Repeated ROLM was performed to completely close the defects. The patient was discharged without adverse events.

**Fig. 1:**
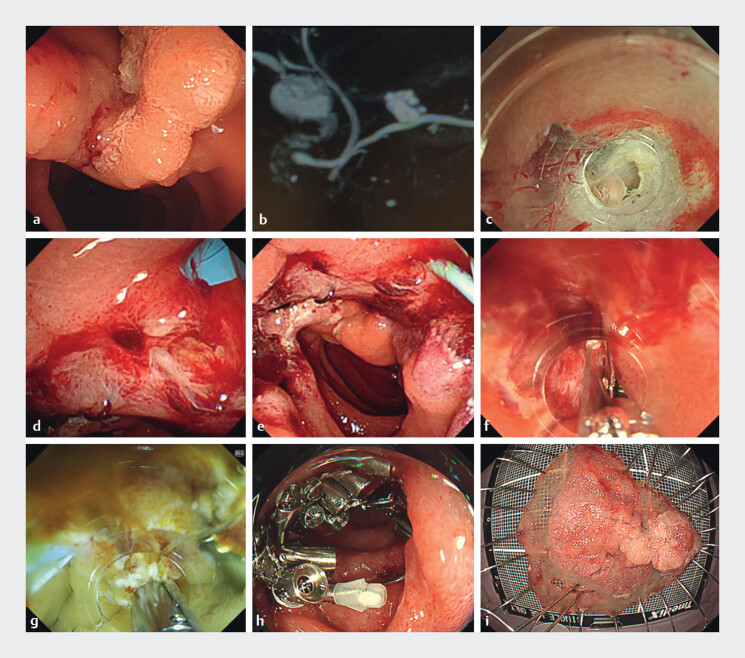
Actual mucosal defect closure using the reopenable-clip over-the-line method (ROLM) after duodenal endoscopic submucosal dissection (ESD) for a minor papilla tumor in a patient with pancreas divisum. **a** Early duodenal cancer near the papilla, completely covering the minor papilla. **b** Partial pancreas divisum shown on magnetic resonance cholangiopancreatography. **c** Opening of the minor papilla during ESD. **d** Insertion of a pancreatic duct stent into the accessory pancreatic duct. **e** The 50-mm ESD defect after complete resection, including the minor papilla. **f** First clip with line placed on the defect edge. **g** Pancreatic duct stent placement confirmed during defect closure. **h** ROLM and pancreatic duct stent after complete defect closure using ESD. **i** The pathological diagnosis of the tumor was intramucosal cancer, and the patient was cured without additional surgery.

Defect closure using the reopenable-clip over-the-line method without plastic stent embedding in the ulcer floor after duodenal endoscopic submucosal dissection for a tumor of the minor papilla in a patient with pancreatic divisum.Video 1

Endoscopy_UCTN_Code_TTT_1AO_2AG_3AD

## Notes

Endoscopy E-Videos https://eref.thieme.de/e-videos
*E-Videos* is an open access online section of the journal *Endoscopy* , reporting on interesting cases and new techniques in gastroenterological endoscopy. All papers include a high-quality video and are published with a Creative Commons CC-BY license. Endoscopy E-Videos qualify for HINARI discounts and waivers and eligibility is automatically checked during the submission process. We grant 100% waivers to articles whose corresponding authors are based in Group A countries and 50% waivers to those who are based in Group B countries as classified by Research4Life (see: https://www.research4life.org/access/eligibility/ ). This section has its own submission website at https://mc.manuscriptcentral.com/e-videos .
